# MicroRNAs secreted by human preimplantation embryos and IVF outcome

**DOI:** 10.1186/s12958-022-00989-0

**Published:** 2022-08-30

**Authors:** Shintaro Kamijo, Toshio Hamatani, Hiroyuki Sasaki, Hiroki Suzuki, Akane Abe, Osamu Inoue, Maki Iwai, Seiji Ogawa, Kei Odawara, Kanako Tanaka, Mutsumi Mikashima, Masami Suzuki, Kenji Miyado, Ryo Matoba, Yasushi Odawara, Mamoru Tanaka

**Affiliations:** 1 Department of Obstetrics and Gynecology, Keio University School of Medicine, 35 Shinanomachi Shinjuku-ku, Tokyo, 160-8582 Japan; 2 Fertility Clinic Tokyo, Tokyo, Japan; 3 Center for Regenerative Medicine, National Center for Child Health and Development (NCCHD), Tokyo, Japan; 4 DNA Chip Research Inc., Tokyo, Japan

**Keywords:** Fertilization in vitro, microRNAs, Gene expression, Embryo culture techniques

## Abstract

**Objective:**

To generate an effective embryo prediction model and identify a non-invasive evaluation method by analyzing microRNAs (miRNAs) in embryo culture medium.

**Design:**

Analysis of microRNA profiles from spent culture medium of blastocysts with good morphology that did or did not result in pregnancy.

**Setting:**

Clinical and experimental research.

**Patients:**

Sixty patients who underwent thawed embryo transfer of blastocysts after intracytoplasmic sperm injection.

**Intervention(s):**

None.

**Main outcome measure(s):**

The association of miRNA abundance levels secreted by blastocysts in culture medium and implantation success.

**Results:**

Our RNA sequencing analysis found a total of 53 differentially expressed miRNAs in the culture media of pregnancy and non-pregnancy groups. Twenty-one miRNAs were analyzed for their potential to predict implantation success. Eight miRNAs (hsa-miR-191-5p, hsa-miR-320a, hsa-miR-92a-3p, hsa-miR-509-3p, hsa-miR-378a-3p, hsa-miR-28-3p, hsa-miR-512-5p, and hsa-miR-181a-5p) were further extracted from the results of a logistic regression analysis of qPCR Ct values. A prediction model for high-quality blastocysts was generated using the eight miRNAs, with an average accuracy of 0.82 by 5-fold cross validation.

**Conclusion:**

We isolated blastocyst miRNAs that may predict implantation success and created a model to predict viable embryos. Increasing the number of investigated cases and further studying the effect of each miRNA on embryonic development is needed to refine the miRNA-based predictive model.

## Capsule

The abundance levels of eight miRNAs (hsa-miR-191-5p, hsa-miR-320a, hsa-miR-92a-3p, hsa-miR-509-3p, hsa-miR-378a-3p, hsa-miR-28-3p, hsa-miR-512-5p, and hsa-miR-181a-5p) secreted by blastocysts in the culture medium can predict implantation success.

## Introduction

Multicellular organisms cooperate with each other by responding to intercellular communication signals, which enables them to maintain homeostasis, growth, proliferation, and reproduction. In addition to direct action by gap junctions, autocrine and paracrine communication, or hormone secretion via blood flow, it has recently been reported that this communication is also maintained by extracellular vehicles (EVs).

EVs themselves were discovered more than 30 years ago and were initially understood as a pathway for the excretion of substances. However, it has become clear that EVs are not just an excretion pathway, but also contribute to intercellular communication by transporting molecules such as lipids, proteins, and nucleic acids (DNA, mRNA, and non-coding RNA), and are used for the exchange of materials between cells. EVs may also play an important role in the reproduction process, particularly in crucial stages such as gametogenesis, fertilization, implantation and embryogenesis [1–3]. In implantation, once the embryo enters the uterine cavity, the embryo and endometrium interact to initiate implantation, and a wide variety of signals such as hormones, growth factors and cytokines are secreted to enable embryo-endometrial crosstalk [4–6].

Some of the transmitters carried by EVs are non-coding RNA, such as micro RNAs (miRNAs), small intervening RNAs (siRNAs), and piwi-interacting RNAs (piRNAs), which play a significant role. miRNAs are the major transcriptional/post-transcriptional regulators of gene expression and are involved in the regulation of diverse biological processes including development, proliferation, differentiation, migration, and apoptosis in vivo. More than 1000 miRNAs have been identified in the human genome.

miRNA expression is dynamically altered during gametogenesis and early embryogenesis in several mammals including cattle [7, 8]. In humans, dynamic changes of miRNAs in cumulus cells, oocytes and follicular fluid have been reported [9–12]. For example, hsa-miR-92a and hsa-miR-130b are highly expressed in follicular fluid from oocytes that failed to fertilize. Follicular fluid from oocytes that became low-grade embryos have high expression levels of hsa-miR-888 and low expression levels of hsa-miR-214 and hsa-miR-454. The miRNA expression level profile depends on the maturation of the oocyte, and influences egg maturation and embryo quality [13, 14].

In reproductive medicine, while single embryo transfer has become a worldwide trend to reduce the risk of multiple births, miscarriage rates are on the rise due to increasing maternal age. This highlights the importance of establishing practical evaluation methods for selecting embryos for transfer. Methods for selecting embryos with the highest potential for development and implantation are critical to maximize the probability of a successful pregnancy. Embryos are generally evaluated by morphological assessment via specular examination, such as using Gardner’s classification. However, this method is not quantitative enough to predict the outcome of implantation because morphological characteristics do not fully reflect embryo integrity. In contrast, preimplantation genetic testing (PGT) that involves invasive procedures, such as biopsies of cleavage stage embryos or trophectoderm cells, are widely used for direct genetic evaluation [15]. PGT for aneuploidy (PGT-A), originally termed preimplantation genetic screening (PGS), aims to detect blastomeres with chromosomal aneuploidies using fluorescent in situ hybridization (FISH). More advanced molecular technologies that use whole genome amplification of trophectoderm cells at the blastocyst stage, such as array comparative genomic hybridization (CGH), single-nucleotide polymorphism (SNP) array and next-generation sequencing (NGS), have further sophisticated PGT-A.

Four randomized trials using blastocyst biopsy and molecular biology techniques have shown improved outcomes in patients over 35 years of age [16–20]. While two of these studies found PGT-A did not improve pregnancy outcomes, they also reported 20–40% of embryos from younger patients had abnormalities [20, 21]. Thus, there is no consensus on the handling of mosaic embryos. Furthermore, there is the possibility that the removal of one or more cells may cause an impairment in the embryo’s developmental potential [22]. Poorly performed blastocyst biopsies can lead to lower implantation rates [23, 24] or create artifactual abnormal PGT results [25, 26]. Therefore, non-invasive methods to evaluate embryo quality are needed to support the diagnosis of chromosome aneuploidy by PGT. Non-invasive PGT-A (niPGT-A), which is performed with DNA collected from the culture medium, has been developed as an effective method for diagnosing aneuploidy and obtaining information about embryo quality and pregnancy outcome. However, in addition to being used for morphological evaluation and chromosome aneuploidy screening, it may also contain biomarkers to assess the quality and implantation potential of the embryo.

Transcriptome and proteome analyses of embryos have a potential clinical applicability and have undergone some progress. miRNAs released from fertilized eggs are rarely secreted in cleavage stage embryos or morulae, but are detected after blastocyst formation via zygotic genome activation (ZGA) [10, 27]. miRNAs secreted by blastocysts in the culture medium can be profiled with high reproducibility [11, 12]. By comparing miRNA profiles against implantation outcomes, it is possible to create a non-invasive model to predict implantation outcomes, which may contribute to the individualization of embryo selection. In this study, we analyzed miRNAs among the non-coding RNAs in the culture medium, with the aim of predicting the implantation outcome of embryos.

## Materials and methods

### Ethical approval and informed consent

This study was approved by the Medical Review Ethics Committee of the Keio University Hospital (#20150473) and written informed consent was obtained from all subjects who agreed to use used embryo culture medium for research purposes.

### Patients

Informed consent was obtained from each participant prior to enrollment. We included 60 patients who underwent thawed embryo transfer of blastocysts after intracytoplasmic sperm injection (ICSI) between May 2016 and April 2019. Patients having undergone conventional in vitro fertilization (IVF) were excluded to avoid contamination of granulosa cells, as were patients with structural and tissue abnormalities or infectious etiologies in the uterus and adnexa. Used embryo culture medium (SAGE 1-Step, Origio) was collected on the fifth day of culture during the IVF cycle. Samples were classified into four groups according to the transferred embryo grade and implantation outcome: I) good morphology blastocyst (*) and positive pregnancy result; II) good morphology blastocyst (*) and negative pregnancy result; III) poor morphology blastocyst (**); and IV) control samples of culture medium only (negative control) (*: Gardner grade ≥ 3BB, **: embryo transfer was not performed for Group III). All patients whose blastocysts had been classified into group II became pregnant within the next three ET cycles, which suggests infertility was not due to uterine conditions.

The mean age of patients whose blastocysts were classified into group I and group II for the RNA sequencing analysis was 36.1 ± 3.6 years (*n* = 12) and 36.7 ± 4.2 years (*n* = 18), respectively. The mean age of patients whose blastocysts were classified into group I, group II, and group III for the RT-qPCR analysis was 34.1 ± 3.2 years (*n* = 17), 36.3 ± 2.8 years (*n* = 17) and 36.2 ± 3.6 years (*n* = 17), respectively. All patients were below 41 years of age.

The stimulation methods were antagonist, short, long, or mild. Ovarian stimulation was initiated on the third day of the menstrual cycle, with 2.5 mg letrozole for 5 days, together with 225 IU of highly purified hMG (Ferring) daily in most cases. A daily dose of 0.25 mg Ganirelix Acetate (Organon) was given when follicle size reached an average diameter of 13–14 mm. Administration of gonadotropin-releasing hormone (GnRH) antagonists was performed when the follicle size was 14 mm or larger. All patients underwent transvaginal ultrasonography and regular monitoring of follicle-stimulating hormone, luteinizing hormone, estrogen, and progesterone. Mature oocytes were collected 34–36 h after human chorionic gonadotropin injection or intranasal GnRH agonist (600 mcg of buserelin, Sanofi) or dual trigger (GnRH agonist plus 250 mcg of choriogonadotropin alfa, Merck Biofarma). Mature oocytes were fertilized in vitro using ICSI methods. All good morphology blastocysts were frozen using the vitrification method. Single embryo transfer (SET) was performed by hormone replacement therapy cycles. If the pregnancy test was positive, follicular hormone and progesterone supplementation were continued until 8 weeks of gestation. If the result was negative, medication was discontinued.

### IVF protocol and sample collection

After ICSI, embryos with two pronuclei were selected by fertilization check. One ICSI embryo each was placed in a microdrop of 30 μL of culture medium (SAGE 1-Step, Origio) sealed with mineral oil and cultured for 5 days in a timelapse incubator (Embryo Scope, Vitrolife) without medium refreshment. When a good blastocyst was obtained, 25 μL of spent medium was collected and cryopreserved. After subsequent thawed embryo transfer, if the outcome was a clinical pregnancy with a fetal heartbeat at ≥ 8 weeks, the spent medium was classified into the pregnancy group (I). In the absence of an increase in hCG blood levels, the medium was classified as belonging to the non-pregnancy group (II). Microdrops of culture medium without embryos were cultured in the same way for 5 days to serve as the control group (IV). Blastocyst grade was evaluated by the Gardner classification just before cryopreservation. The same manufacturer lot of culture medium was used for all groups.

### Library preparation and next-generation sequencing

To investigate the biological relationship between miRNA expression profiles and successful implantation, RNA sequencing was performed on miRNAs in the pregnancy and non-pregnancy groups (I and II), as well as in the control group (IV) to correct for the presence of non-specific background miRNAs, including those derived from serum albumin in the culture medium. Among the non-coding RNAs obtained, the percentage of miRNAs was very small, about 0.04–0.2%. To ensure sufficient miRNA abundance, three pooled samples each were prepared by combining four and six culture media in group I and II, respectively.

In PCR-based whole amplification, 30 cycles of PCR amplification of the DNA library with adapters allowed sequencing. The miRNA sequencing library was constructed using the TruSeq® Small RNA Sample Prep Kit according to the manufacturer’s protocol (Illumina, San Diego, CA, USA). To the total RNA of each sample we added miRNAs (dme-miR-308-3p and dme-miR-316-5p) that were spiked as carrier RNA, to prepare the miRNA sequencing library in the following steps: 3′-adaptor ligation, 5′-adaptor ligation, cDNA synthesis, PCR amplification and AMPure beads (Beckman Coulter, Brea, CA, USA) purification for PCR-amplified fragments. After quantification with RT-PCR, the libraries were captured on cBOT (Illumina, San Diego, CA, USA) to be amplified in situ as clusters. Finally, they were sequenced on the HiSeq™ 1500 System (Illumina, San Diego, CA, USA) as per the manufacturer’s instructions.

After sequencing, the adaptor sequences were trimmed and removal of non-small RNA-related reads such as null-insert (insert size < 10 nt), 5′ adapter contamination and poly-A containing, were performed by custom perl scripts. The trimmed reads were aligned to the human reference genome (hg19) using COBWeB aligner implemented in StrandNGS ver. 2.6 (Agilent Technologies, Santa Clara, CA). The UCSC transcripts ver.2013.12.3 dataset was used for gene annotation. Up to one mismatch per each sequencing read was allowed in the alignment. The raw read count values estimated for each small RNA were normalized using the Trimmed Mean of M-value (TMM) method [28].

### RT-qPCR

The spent medium obtained from each embryo was analyzed separately without pooling and miRNAs secreted or absorbed from each embryo were quantitatively analyzed by quantitative RT-PCR. We measured Ct values in 17 samples in the good morphology/pregnancy group (I), 17 samples in the good morphology/non-pregnancy group (II), 17 samples in the poor morphology (III), and 11 samples in the control group (IV) using only culture medium. The TaqMan Advanced miRNA cDNA Synthesis Kit (ThermoFisher) was used to synthesize cDNA according to the manufacturer’s protocol, followed by pre-amplification. To 5 uL of pre-amplified cDNA we added 10 uL of TaqMan Fast Advanced Master Mix (2X), 1 uL of TaqMan Advanced miRNA Assay (20X), and RNase free water to make 20 uL. Then, the PCR was run with 40 cycles of enzyme activation at 95 °C for 20 s, one denaturation cycle of 95 °C for 3 s, and an anneal/extend stage at 60 °C for 30 s, and the fluorescence intensity of FAM was detected with a 7500 real-time PCR system (ThermoFisher). Normalization was performed using pre-spiked *C. elegans*-derived miRNA as a control gene.

### Data analysis

Obtained sequence data was trimmed and aligned to the human genome sequence using the COBWeb aligner tool (Agilent Technologies, Santa Clara, CA, USA). miRNA annotation information was obtained from UCSC ver2013.12.3 database. Alignments were allowed under single nucleotide mismatches, and normalization was performed for raw read counts using the Trimmed Mean of M-value (TMM) method [27].

### Logistic regression analysis

A prediction model was generated based on the results from expression levels of indicated miRNAs by logistic regression analysis to predict implantation outcome. A ROC curve was generated by plotting the true positive rate against the false positive rate, which was calculated by comparing the predicted value using the prediction model with the true result. Five-fold cross validation was used to validate the generalization performance of the model. Each dataset was randomly divided into five subsets of equal size, and the model was trained using four of the five subsets and validated on the remaining holdout subset. All combinations were performed, and the AUC was calculated for each validation and the final AUC was reported as the average AUC of five individual training trials.

### Target prediction and functional analysis

We performed a web-based database search for miRNAs indicated to be involved in the implantation process. The databases used were DIANA miRPath-v3.0 (http://www.microrna.gr/miRPathv3) and miRTargetLink Human (https://ccb-web.cs.uni-saarland.de/mirtargetlink). These databases give an overview of miRNAs and provide information on the predicted target genes and their targets.

## Results

### RNA sequencing

To determine if the abundance of miRNAs in the embryo culture medium correlated with the outcome of the subsequent implantation of each blastocyst, we compared the expression of miRNAs between the pregnancy (I) and non-pregnancy (II) groups, the pregnancy (I) and control (IV) groups, and the non-pregnancy (II) and control (IV) groups. We calculated *P*-values with a T-test using the asymptotic method and selected miRNAs with a fold change > 2 and *P*-value < 0.05, respectively.

**Fig. 1:**
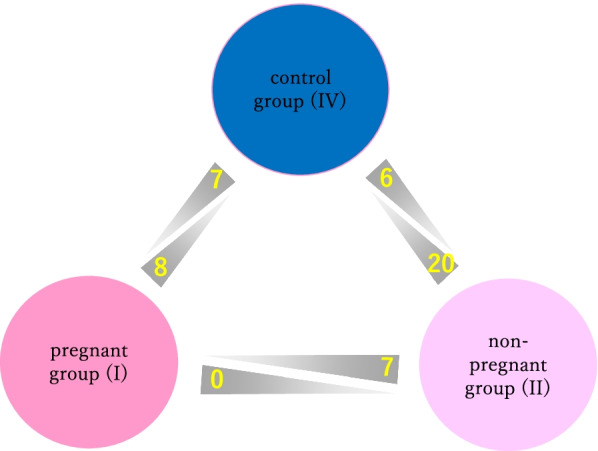
Seven miRNAs were more abundant in the non-pregnancy group (II) than in the pregnancy group (I). Seven miRNAs were more abundant in the control group (IV) than in the pregnancy group (I), and eight miRNAs were more abundant in the pregnancy group (I) than in the control group (IV). Six miRNAs were more abundant control group (IV) than in the non-pregnancy group (II) and 20 miRNAs were more abundant in the non-pregnancy group (II) than in the control group (IV)

We identified 48 miRNAs that showed significant expression differences (Table 1). These included miR-16, miR-191 and miR-192, which are generally thought to be present in serum as circulating miRNAs [29–31]. The miRNAs that showed significant differences in expression levels between the groups [pregnancy group (I), non-pregnancy group (II), and control group (IV)] are shown in Fig. 1.

**Table 1:** RNA sequencing analysis of miRNAs in spent blastocyst culture medium

Comparison	miRNA	Regulation	Fold Change	***P*** value
I vs II	hsa-miR-320a	down	120.27	< 0.001
	hsa-miR-320b	down	81.82	< 0.001
	hsa-miR-30e-5p	down	76.80	0.008
	hsa-let-7c-5p	down	75.90	0.003
	hsa-miR-99b-5p	down	39.22	0.011
	hsa-miR-16-5p	down	32.20	0.012
	hsa-miR-509-3p	down	23.60	0.048
I vs IV	hsa-miR-320b	down	248.13	< 0.001
	hsa-miR-320a	down	238.04	< 0.001
	hsa-miR-203a	down	20.88	0.019
	hsa-miR-184	down	4.94	0.005
	hsa-miR-1910-5p	down	2.79	0.047
	hsa-miR-486-5p	down	2.55	0.005
	hsa-miR-320c	down	2.45	0.044
	hsa-miR-148a-3p	up	90.04	< 0.001
	hsa-miR-371a-5p	up	88.41	0.004
	hsa-miR-192-5p	up	85.39	< 0.001
	hsa-miR-28-3p	up	75.82	< 0.001
	hsa-miR-378a-3p	up	74.27	< 0.001
	hsa-miR-373-3p	up	65.71	< 0.001
	hsa-let-7i-5p	up	52.62	< 0.001
	hsa-miR-181a-5p	up	14.11	0.026
II vs IV	hsa-miR-184	down	7.62	0.001
	hsa-miR-203a	down	6.81	0.017
	hsa-miR-486-5p	down	3.85	0.001
	hsa-miR-320b	down	3.03	0.048
	hsa-miR-1910-5p	down	2.56	0.001
	hsa-miR-320c	down	2.45	0.044
	hsa-miR-371a-5p	up	294.07	< 0.001
	hsa-miR-191-5p	up	178.42	< 0.001
	hsa-miR-192-5p	up	172.80	< 0.001
	hsa-miR-373-3p	up	165.56	< 0.001
	hsa-miR-378a-3p	up	160.10	< 0.001
	hsa-miR-16-5p	up	158.94	< 0.001
	hsa-let-7c-5p	up	136.09	< 0.001
	hsa-miR-372–3p	up	115.09	< 0.001
	hsa-miR-148a-3p	up	114.29	< 0.001
	hsa-miR-28-3p	up	114.13	< 0.001
	hsa-let-7i-5p	up	103.90	0.002
	hsa-miR-509-3p	up	88.80	< 0.001
	hsa-miR-146b-5p	up	88.36	< 0.001
	hsa-miR-30e-5p	up	65.59	0.009
	hsa-miR-21-3p	up	64.15	< 0.001
	hsa-let-7a-5p	up	51.99	< 0.001
	hsa-let-7b-5p	up	41.52	< 0.001
	hsa-miR-99b-5p	up	40.72	0.010
	hsa-miR-92a-3p	up	31.57	0.038
	hsa-miR-181a-5p	up	26.31	0.011

Forty-eight miRNAs were differentially expressed in spent culture media, when comparing the pregnancy group (I) with the non-pregnancy group (II), the pregnancy group (I) with the control group (IV), or the non-pregnancy group (II) with the control group (IV). A t-test was used to calculate P-values using the asymptotic method, and the number of miRNAs that met fold change > 2 and a P-value < 0.05 were calculated

Seven miRNAs were identified as differentially expressed miRNAs in the non-pregnancy group (II) compared with the pregnancy group (I): hsa-miR-16-5p, hsa-miR-30e-5p, hsa-miR-320a, hsa-miR-320b, hsa-miR-509-3p, hsa-miR-99b-5p and hsa-let-7c-5. In contrast, no miRNAs were expressed more highly in the pregnancy group (I) compared with the non-pregnancy group (II).

When comparing the pregnancy (I) and control (IV) groups, the most frequently expressed miRNAs in the pregnancy group were hsa-miR-148a-3p, hsa-miR-181a-5p, hsa-miR-192-5p, hsa-miR-28-3p, hsa-miR-371a-5p, hsa-miR-378a-3p, hsa-miR-373-3p and hsa-let-7i-5p. The seven most frequently expressed in the control group compared with the pregnancy group (I) were hsa-miR-184, hsa-miR-1910-5p, hsa-miR-203a, hsa-miR-320a, hsa-miR-320b, hsa-miR-320c and hsa-miR-486-5p.

We identified 20 miRNAs expressed more frequently in the non-pregnancy group compared with the control group. In contrast, in the control group, six miRNAs (hsa-miR-184, hsa-miR-1910-5p, hsa-miR-203a, hsa-miR-320b, hsa-miR-320c and hsa-miR-486-5p), were highly expressed. Similar results were observed when comparing pregnancy and control groups except for hsa-miR-320a. Furthermore, hsa-miR-320a is a miRNA that is more frequently expressed in the non-pregnancy group than in the pregnancy group.

### RT-qPCR

The expression of these miRNAs in the spent medium of individual blastocysts was confirmed by relative quantitative real-time PCR (RT-qPCR). In addition to the 15 miRNAs that showed significant expression differences between the three groups in the RNA sequencing analysis, we also studied the expression of five miRNAs that have been suggested to be involved in embryonic maturation and implantation [10, 12, 32, 33]. The Ct values are shown in Table 2.

**Table 2:** Quantitative RT-PCR on miRNAs in spent blastocyst culture medium

	miRNA	Group I	Group II	Group III	Group IV
Internal standard gene	cel-miR-39				
ΔCt	hsa-let-7c-5p	12.0	12.0	11.0	11.9
	hsa-let-7i-5p	8.4	8.4	7.9	8.5
	hsa-miR-148a-3p	10.7	9.1	6.4	10.3
	hsa-miR-16-5p	9.5	9.9	5.7	16.4
	hsa-miR-181a-5p	7.5	8.8	8.9	10.6
	hsa-miR-191-5p	6.5	6.7	3.7	6.9
	hsa-miR-192-5p	3.7	3.8	2.9	3.6
	hsa-miR-30e-5p	−5.3	−6.0	−6.8	−6.1
	hsa-miR-320a	6.9	7.6	3.5	9.4
	hsa-miR-92a-3p	5.8	5.9	2.5	7.9
	hsa-miR-99b-5p	10.3	10.5	12.5	20.9
	hsa-miR-25-3p	8.4	8.4	4.5	9.9
	hsa-miR-509-3p	0.0	−0.2	−0.7	−0.6
	hsa-miR-378a-3p	9.6	9.5	4.7	10.0
	hsa-miR-372–3p	4.5	5.6	−1.3	NaN
	hsa-miR-28-3p	10.6	10.2	6.8	11.0
	hsa-miR-371a-5p	7.6	9.6	3.2	NaN
	hsa-miR-373-3p	5.2	6.4	−0.6	9.7
	hsa-miR-512-5p	5.9	5.9	2.1	6.5
	hsa-miR-320b	8.6	9.4	6.0	10.8

In addition to the 15 miRNAs that showed significantly different expression levels between the groups in the RNA sequencing analysis, five miRNAs suggested to contribute to embryonic maturation and implantation in literature were assessed. Their expression in the culture medium of individual blastocysts was analyzed using relative quantitative analysis by Taqman realtime PCR. We found that embryos with good morphology showed higher ΔCt values than those with poor morphology, and that the pregnancy group tended to show higher ΔCt values than the non-pregnancy group

**Fig. 2:**
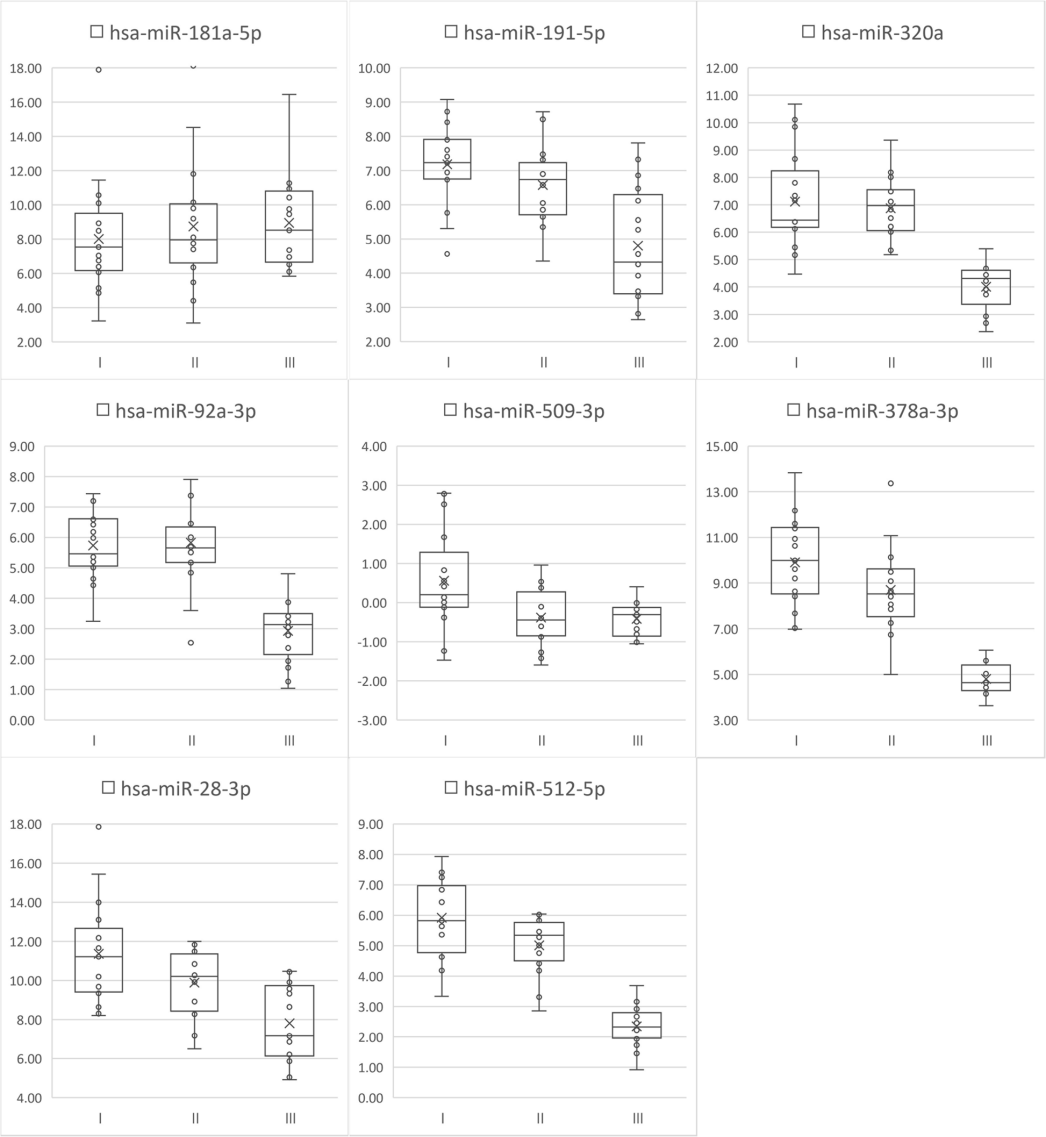
The abundance levels of the eight miRNAs used for the ROC curve in spent culture media from embryos with different grades and IVF outcomes, as determined by RT-qPCR analysis. A vertical axis represents ΔCt

Comparing groups I/II with III using Tukey’s honestly significant difference test, we found that embryos with good morphology tended to show higher ΔCt values than those with poor morphology. For example, hsa-miR-512-5p showed significantly different ΔCt values for all combinations of I/II, I/III, and II/III, and hsa-miR-509-3p showed significant differences for combinations of I/II and I/III, and hsa-miR-191-5p, hsa-miR-320a, hsa-miR-92a-3p, hsa-miR-378a-3p and hsa-miR-28-3p showed significant differences for combinations of I/ III and II /III (Fig. 2).

### In-silico analysis

Prediction performance of implantation outcome was confirmed using logistic multiple regression analysis based on RT-qPCR results. When all miRNAs were analyzed, the median accuracy of the 5-fold cross validation for the logistic regression analysis was 0.78.

**Fig. 3:**
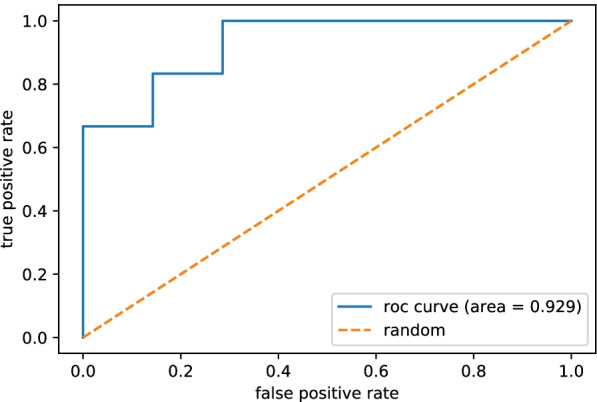
Receiver operating characteristic curves of the validated abundant miRNAs as determined by single reverse-transcription quantitative polymerase chain reaction validation analysis

We selected eight miRNAs (hsa-miR-191-5p, hsa-miR-320a, hsa-miR-92a-3p, hsa-miR-509-3p, hsa-miR-378a-3p, hsa-miR-28-3p, hsa-miR-512-5p and hsa-miR-181a-5p) demonstrating a high contribution to implantation from the logistic regression analysis plus another five miRNAs expected to have a significant contribution to implantation according to literature. We performed a logistic regression analysis on these eight miRNAs to generate ROC curves for each miRNA alone and in combination with the other miRNAs (Fig. 3).

## Discussion

Preimplantation development involves events such as zygotic genome activation (ZGA), which generates a totipotent state of each blastomere at the cleavage stage; and first lineage differentiation, which gives rise to the inner cell mass (ICM) and the trophectoderm at the blastocyst stage [27]. These are achieved by spatiotemporal-specific expression of huge numbers of zygotic genes regulated by DNA methylation, histone modification, ATP-dependent chromatin remodeling and microRNAs [34–37].

In this study, we focused on the importance of miRNA expression during preimplantation development and tried to utilize microRNA profiles in spent blastocyst culture medium to diagnose embryo quality. We used RNA sequencing and RT-qPCR to analyze the miRNA profiles of spent media in which ICSI embryos were cultured. RNA sequencing results identified 48 differentially expressed miRNAs between the groups, with a large number of miRNAs significantly more highly expressed in the non-pregnancy group compared with the pregnancy and control groups. These results suggest that miRNAs are secreted by the embryo, but more miRNAs are overexpressed in embryos that do not result in a viable pregnancy. Since it has been suggested that healthy embryos are metabolically quiescent [38], pathological conditions that compromise the quality of the embryo may require compensatory expression of more microRNAs.

For further investigation, we selected seven miRNAs that were more abundant in the non-pregnancy group than in the pregnancy group, and eight miRNAs that were more abundant in the pregnancy group than in the control group. We also added five miRNAs that may play a fundamental role in embryonal development according to literature: hsa-miR-191, which has been detected abundantly in medium containing aneuploid embryos [12], hsa-miR-92a, which is highly expressed in the follicular fluid from oocytes that failed to fertilize [13], hsa-miR-372, which is more abundant in euploid embryos but also in failed IVF cases [12, 32], has-miR-25, which has been detected more frequently in media containing degenerate embryos [33], and has-miR-512, which has been detected more frequently in media containing blastocysts that go on to implant successfully [10]. The expression levels of 20 miRNAs in the culture medium of individual blastocysts were examined using RT-qPCR. We have identified biomarkers specific for group III that seldom overlapped with group I and II. This result means that these miRNAs may be useful to segregate embryos with good or poor morphology. With the significant overlap in biomarkers between groups I and II, there is an opportunity to improve this particular miRNA-based diagnostic method to evaluate the possibility of pregnancy.

Based on the RT-qPCR results, we validated the prediction performance by logistic multiple regression analysis and extracted eight miRNAs (hsa-miR-181a-5p, hsa-miR-191-5p, hsa-miR-320a, hsa-miR-92a-3p, hsa-miR-509-3p, hsa-miR-378a-3p, hsa-miR-28-3p and hsa-miR-512-5p), which are predicted to influence implantation success. Analysis of the bioinformatics database suggested that hsa-miR-92a-3p, hsa-miR-509-3p, hsa-miR-191-5p and hsa-miR-181a-5p are involved in cell adhesion, hsa-miR-378a-3p and hsa-miR-512-5p are involved in oocyte meiosis, and hsa-miR-181a-5p is involved in the estrogen signaling pathway.

Hsa-miR-181a-5 was abundant in the pregnancy group, while all the remaining seven microRNAs were more expressed in the non-pregnancy group. As discussed in literature, miR-181a is an important regulator of CARM1 in mouse preimplantation embryos [38, 39]. CARM1 is a type I arginine methyltransferase involved in methylation of histone H3R26 and regulates cell division and differentiation during mouse oogenesis and preimplantation development. The endonuclear distribution of CARM1 protein, which binds to the long non-coding RNA *Neat1* and forms paraspeckles, is heterogeneously expressed between blastomeres from the four-cell stage to the blastocyst stage [40]. Accordingly, the heterogeneity of CARM1 distribution leads to the differentiation into inner cell mass and trophectoderm by regulating gene expression of Oct4, Sox2, Nanog and Cdx2 [40]. In this study, hsa-miR-181a tended to be highly expressed in the pregnancy group, and hsa-miR-181a was inferred to contribute to healthy embryonal development by participating in the control of heterogeneous CARM1 distribution.

In contrast, it has previously been reported that miR-191 was more highly concentrated in media from aneuploid embryos, and miR-191, miR-372 and miR-645 were more highly concentrated in media from failed IVF cycles [12]. In a study that contrasted the grade of embryos at the cleavage stage and implantation outcome with miRNAs in culture medium and sperm, it was reported that miR-320a, as well as miR-19b-3p, miR-15a-5p, miR-21-5p and miR-20a-5p miRNAs were differentially expressed [41]. A study comparing the expression of miRNAs in human follicular fluid showed that miRNA-320a levels were significantly different in top-quality embryos versus non-top-quality embryos on day 3, and miR-378-3p had a higher abundance level from patients treated with human menopausal gonadotropin (hMG) than those treated with recombinant follicle-stimulating hormone (rFSH) [42]. In this study, the expression of miR-320a was also different between pregnancy and non-pregnancy groups.

We aimed to identify miRNAs that are released from the cells via EVs and analyze their relationship with embryo maturation and implantation. miRNAs are not chromosomes or genes themselves, but they are the most important substantial regulators of post-transcriptional gene regulation. Understanding miRNA biogenesis, function, and mechanisms of regulating gene expression is important to elucidate the complex gene regulatory network during embryonic development. The several miRNAs we identified as embryonal markers for implantation using logistic regression analysis in this paper are well validated in literature. The next step will be to elucidate the molecular functions of the eight miRNAs in relation to implantation or peri-implantation embryonal development. In conclusion, miRNA profiling of the culture medium may be a useful and feasible assay to support morphological evaluation and chromosome aneuploidy screening, since it is non-invasive and culture medium is easy to collect.

## Data Availability

The data that support the findings of this study are available from the corresponding author, S.K., upon reasonable request.
